# Hydrogen Sulfide Reduces Myeloid-Derived Suppressor Cell-Mediated Inflammatory Response in a Model of *Helicobacter hepaticus*-Induced Colitis

**DOI:** 10.3389/fimmu.2018.00499

**Published:** 2018-03-27

**Authors:** Paola De Cicco, Theodore Sanders, Giuseppe Cirino, Kevin J. Maloy, Angela Ianaro

**Affiliations:** ^1^Department of Pharmacy, University of Naples Federico II, Naples, Italy; ^2^Sir William Dunn School of Pathology, University of Oxford, Oxford, United Kingdom

**Keywords:** colitis-associated cancer, colorectal cancer, cystathionine gamma-lyase, cystathionine beta-synthase, *Helicobacter hepaticus*, hydrogen sulfide, myeloid-derived suppressor cells

## Abstract

Chronic inflammation contributes to tumor initiation in colitis-associated colorectal cancer (CRC). Indeed, inflammatory bowel disease (IBD) patients show an increased risk of developing CRC. Cancer immune evasion is a major issue in CRC and preclinical and clinical evidence has defined a critical role for myeloid-derived suppressor cells (MDSCs) that contribute to tumor growth and progression by suppressing T-cells and modulating innate immune responses. MDSCs comprise a heterogeneous population of immature myeloid cells that can be distinct in two subtypes: CD11b^+^Ly6G^+^Ly6C^low^ with granulocytic phenotype (G-MDSCs) and CD11b^+^Ly6G^−^Ly6C^high^ with monocytic phenotype (M-MDSCs). Hydrogen sulfide (H_2_S) is an endogenous gaseous signaling molecule that regulates various physiological and pathophysiological functions. In particular, several studies support its anti-inflammatory activity in experimental colitis and ulcer. However, the role of the H_2_S pathway in innate immune-mediated IBD has not yet been elucidated. To better define a possible link between MDSCs and H_2_S pathway in colitis-associated CRC development, we used an innate immune-mediated IBD model induced by infection with the bacterium *Helicobacter hepaticus* (*Hh*), closely resembling human IBD. Here, we demonstrated an involvement of MDSCs in colitis development. A significant time-dependent increase of both G-MDSCs and M-MDSCs was observed in the colon and in the spleen of *Hh*-infected mice. Following, we observed that chronic oral administration of the H_2_S donor DATS reduced colon inflammation by limiting the recruitment of G-MDSCs in the colon of *Hh*-infected mice. Thus, we identify the metabolic pathway l-cysteine/H_2_S as a possible new player in the immunosuppressive mechanism responsible for the MDSCs-promoted colitis-associated cancer development.

## Introduction

Colorectal cancer (CRC) is one of the major cause of morbidity and mortality throughout the world. It is the third most common cancer worldwide^1^. Chronic intestinal inflammation is the primary risk factor for the development of gastrointestinal malignancy (1). A meta-analysis estimates the risk of CRC in ulcerative colitis patients to be 2% after 10 years, 8% after 20 years, and 18% after 30 years of disease (2). Although the exact etiology remains unclear, a multifactorial interaction among immunological, genetic, and environmental factors contribute to the disturbance of homeostasis leading to the generation of an abnormal immune response against the commensal microbiota (3, 4). In particular, loss of the intestinal homeostasis and induction of pathogenic inflammatory response is first related to the aberrant activation of the innate immune system in the gut that consists of intestinal epithelial cells and several leukocyte population (i.e., neutrophils, dendritic cells, monocytes/macrophages, and innate lymphoid cells) (5). Cancer immune evasion is a major issue in CRC and preclinical and clinical evidence has defined a critical role for myeloid-derived suppressor cells (MDSCs) in modulating the innate immune responses by suppressing T cell anti-tumor functions (6). MDSCs consist of a heterogeneous population of immature myeloid cells characterized by co-expression of CD11b and Gr-1 (7). Two distinct subtypes of MDSCs have been identified in tumor-bearing mice: CD11b^+^Ly6G^+^Ly6C^low^ with granulocytic phenotype (G-MDSCs) and CD11b^+^Ly6G^−^Ly6C^high^ with monocytic phenotype (M-MDSCs). Both M-MDSCs and G-MDSCs exercise their potent immunosuppressive activity by modifying the microenvironment through the depletion of amino acids (arginine, tryptophan, glutamine, and cysteine). So far, the main immunosuppressive mechanisms described are based on l-arginine metabolism *via* Arginase 1 (ARG1) and inducible nitric oxide synthase 2 (NOS2) and reactive oxygen species production (8–10). In CRC, the blood MDSCs numbers correlate with stage and metastatic burden (11). Similar G-MDSCs and M-MDSCs populations have also been described in inflammatory bowel diseases (IBD). However, their role in both IBD and CRC still needs to be elucidated (12).

*Helicobacter* species that colonize the lower bowel and biliary tract of mice have been associated with the development of colitis resembling human IBD in susceptible hosts. Furthermore, *Helicobacter hepaticus* (*Hh*) infection has been demonstrated to exacerbate the development of cancer at both intestinal and extra-intestinal sites (13). To better define the role of MDSCs in colitis development, we used an innate immune-mediated IBD model induced by infection with *Hh*. Epithelial hyperplasia and crypt abscesses, associated with a marked granulocyte accumulation within intestinal tissues, characterize this preclinical model closely resembling the human IBD (14). *Hh* is a gram-negative, spiral-shaped, microaerophilic bacterium that is a common member of the mouse intestinal microbiota found predominantly in the cecum and colon. Although it does not cause invasive infections in most immune competent mouse strains, *Hh* induces chronic intestinal inflammation in susceptible mice lacking adaptive immune system, such as 129SvEvRag^−/−^ mice, that can eventually progress to colon cancer (13).

Recently, it has been demonstrated that hydrogen sulfide (H_2_S) promotes the resolution of colitis and enhance ulcer healing (15, 16). H_2_S is an endogenous gaseous signaling molecule that regulates various physiological and pathophysiological functions. In particular, H_2_S exhibits several anti-inflammatory effects such as reduction of edema formation and suppression of pro-inflammatory cytokines release (17). In mammals, H_2_S is endogenously produced from sulfur-containing amino acids, such as l-cysteine (l-cys), mainly by two pyridoxal-5′-phosphate (P5P)-dependent enzymes, cystathionine gamma-lyase (CSE, EC 4.4.1.1), and cystathionine beta-synthase (CBS, EC4.2.1.22). H_2_S is also generated from dietary sulfate metabolism in the lumen of the large intestine by anaerobic sulfate-reducing bacteria (18). The role of H_2_S colonic inflammation and cancer has been recently reviewed (18–20). However, the role of hydrogen sulfide pathway in innate immune-mediated IBD has not yet been elucidated. Here, we have evaluated the role of MDSCs and its link with the hydrogen sulfide pathway in the intestinal inflammation development upon stimulation with pathogenic *Hh*.

## Materials and Methods

### Animals

*129SvEvRag2^−/−^* (*Rag2^−/−^*) mice were bred and maintained under specific pathogen-free conditions in an accredited animal facility in the Pathology Services Building at the University of Oxford. Experiments were conducted in accordance with the UK Scientific Procedures Act (1986) under a Project License (PPL) authorized by the UK Home Office Animal Procedures Committee and approved by the Sir William Dunn School Ethical Review Committee.

### Induction of Colitis

*Helicobacter hepaticus* NCI-Frederick isolate 1A (strain 51449) was grown on blood agar plates containing trimethoprim, vancomycin, and polymyxin B under microaerophilic conditions. Cultures were expanded for 3–4 days in TSB (Oxoid) containing 10% FCS (GIBCO BRL) and *Helicobacter*-free *Rag2^−/−^* mice were fed three times on consecutive days with *Hh* 1A (1.0 × 10^8^ CFU) by oral gavage. Mice were sacrificed at different time points (3 and 6 weeks) after the first *Hh* inoculation.

### *In Vivo* Drug Treatment

*Helicobacter hepaticus*-infected Rag2^−/−^ mice received diallyl trisulfide (DATS; 50 mg/kg) or vehicle (PBS) starting at week 4 after the first *Hh* inoculation. DATS or vehicle were given by oral gavage once a day for 14 days. Treatment groups were mixed in cages to minimize cage effects.

### Assessment of Intestinal Inflammation

Mice were sacrificed when symptoms of clinical disease (diarrhea) became apparent in control groups, usually 6 weeks after initiation of experiments. Samples of proximal, mid, and distal colon were immediately fixed in buffered 10% formalin. Four to five microns of paraffin-embedded sections were stained with hematoxylin and eosin, and inflammation was graded according to the following scoring system. Each sample was graded semi-quantitatively from 0 to 3 for four criteria: (1) degree of epithelial hyperplasia and goblet cell depletion; (2) leukocyte infiltration in the lamina propria; (3) area of tissue affected; (4) presence of markers of severe inflammation such as crypt abscesses, submucosal inflammation, and ulcers. Typical features of each grade are as follows: 0 = normal; 1 = mild epithelial hyperplasia; 2 = pronounced hyperplasia with substantial leukocytic infiltrates; 3 = severe hyperplasia severe transmural inflammation, ulceration, crypt abscesses, and severe depletion of goblet cells. Scores for each criterion were added to give an overall inflammation score for each sample of 0–12. The individual scores from the section of proximal, mid, and distal colon were averaged to obtain the total inflammation scores for the colon.

### Isolation of Leukocyte Subpopulations from Spleen and Colon

Cell suspensions from spleen and colon from infected and uninfected *Rag2^−/−^* mice were prepared as described below. Colons were longitudinally opened, cut into 1-cm pieces, and incubated (three times) in RPMI 1640 with 10% FCS and 5 mM EDTA at 37°C to remove epithelial cells. Tissue was then digested with collagenase VIII/DNase I solution for 45 min at 37°C. The isolated cells were layered on a 30/40/75% Percoll gradient, which was centrifuged for 20 min at 600 g, and the 40/75% interface, containing mostly leukocytes, was recovered. Spleens were crushed in petri dish on filter with syringe. The cells were collected and suspended in ACK for the lysis of red blood cells and then centrifuge at 1,500 rpm for 5 min. Cells were then analyzed using flow cytometry.

### Flow Cytometry

Aliquots of 5 × 10^5^ cells were washed in FACS buffer (PBS, 0.1% BSA), incubated with a fixable viability dye and anti-Fc receptor (αCD16/32), and stained using the following panel of monoclonal antibodies to murine cell surface molecules (all from BD Biosciences): PerCP-Cy5.5-conjugated anti-CD11b, PE-conjugated anti-Ly6G, FITC-conjugated anti-Ly6C, and Violet1-conjugated anti-CD45. Cells were washed in FACS buffer and analyzed by Dako Cyan Flow cytometry.

### H_2_S Synthesizing Activity in Colon

Samples from colon of 1 cm from infected and uninfected *Rag2^−/−^* mice were taken from the proximal, mid, and distal region, snap frozen in liquid nitrogen and stored at −80°C until the H_2_S assay was performed. Colon pieces were homogenized in ice-cold 100 mmol/L potassium phosphate buffer (pH = 7.4), sodium orthovanadate 10 mM, PMSF 100 mM, and protease inhibitor cocktail (Sigma-Aldrich, St. Louis, MO, USA) with a FastPrep™24 homogenizer, and the protein concentration was determined using the Bradford assay. H_2_S synthesis from colon tissue homogenates was measured in the presence of the exogenous substrate l-cys and the cofactor required by H_2_S-producing enzymes, P5P and in the presence of the CSE inhibitor (dl-propargylglycine; PAG) or CBS inhibitor (O-carboxymethyl-hydroxylamine hemihydrochloride, CHH). The lysates were added in a reaction mixture (total volume 500 µL) containing P5P (2 mM, 20 µL), l-cys (10 mM, 20 µL), and saline (30 µL) or P5P (2 mM, 20 µL), L-cys (2 mM, 20 µL), and CHH/PAG (30 µL). The reaction was performed in parafilm-sealed Eppendorf tubes and initiated by transferring tubes from ice to a 37°C water bath. After 40 min incubation, zinc acetate 1% (ZnAc; 250 µL) was added to trap any H_2_S emitted followed by trichloroacetic acid 10% (TCA; 250 µL). Subsequently, *N*,*N*-dimethylphenylendiamminesulphate 20 µM (DPD; 133 µL) in 7.2 M HCl and FeCl_3_ (30 µM, 133 µL) in 1.2 M HCl were added. After 20 min, absorbance values was measured at 670 nm with a microplate reader and H_2_S concentration was calculated against a calibration curve of NaHS (3.12–250 µM). Results are expressed as nanomoles per milligram protein per minute.

### Immunoblot

Cystathionine beta-synthase and CSE intestinal levels was performed by Western blot analysis of colonic tissues of either healthy mice or mice 6 weeks following the induction of colitis. Tissue homogenates were obtained as described above. Equal amounts of protein (40 µg) were loaded onto a 10% gel, subjected to SDS-PAGE, and electro-transferred onto polyvinylidene difluoride (PVDF) membranes (Hybond-P PVDF Membrane, Amersham Biosciences, Buckinghamshire, UK). The membranes were blocked for 2 h in 5% low-fat milk in PBS with 0.1% Tween 20 (PBST) at room temperature. Then, the filters were incubated with the following primary antibodies: CSE (1:500 dilution, Proteintech), CBS (1:500, Novus Biological), and GAPDH (1:1,000 dilution, Santa Cruz Biotechnology) overnight at 4°C. The membranes were washed three times with PBST and then incubated with HRP-conjugated anti-mouse or anti-rabbit IgG (1:2,000, Cell Signaling) for 2 h at room temperature. The immune complexes were detected by the ECL chemiluminescence method (Thermo Fisher Scientific).

### RNA Purification and Quantitative Real-Time PCR

Colon samples were snap frozen in liquid nitrogen. Tissue material was homogenized in RLT buffer (QIAGEN) with β-mercaptoethanol using FastPrep™24 homogenizer (MP Biomedicals) with lysing matrix D beads (MP Biomedicals). RNA isolation was performed using the RNeasy kit (QIAGEN). RNA purity and quantification was determined using a Nanodrop spectrophotometer (Thermo Fisher Scientific). cDNA synthesis was performed using the Superscript III reverse transcription kit from Invitrogen. Quantitative real-time PCR for the candidate genes was carried out as described before (21) using the following primer pairs: CBS isoform 1: 5′-CCAGGCACCTGTGGTCAAC-3′ and 3′-GGTCTCGTATTGGATCTGCT-5′; CSE: 5′-TTCCTGCCTAGTTTCCAGCAT-3′, and 3′-GGAAGTCCTGCTTAAATGTGGTG-5′; IL-6: 5′-GAGGATACCACTCCCAACAGACC-3′ and 3′-AAGTGCATCATCGTTGTTCATACA-5′; TNF-α: 5′-TACTGAACTTCGGGGTGATTGGTCC-3′ and 3′-CCTGGTTAGTGGGGCTTCAAGTCAT-5′. Primers for Hprt (Mm01545399_m1), was obtained from Applied Biosystems. cDNA samples were analyzed in triplicate and values were normalized on Hprt expression. Analysis was performed according to the ∆−Ct method.

### Quantification of *Hh* in Cecal Contents

DNA was purified from cecal content taken from H*h*-infected mice using the DNA Stool kit (QIAGEN). *Hh* DNA was quantified using a real-time PCR method based on the cdtB gene, performed with the ABI prism Taqman 7700 sequence detection system (PE Biosystems). Standard curves were constructed using *Hh* DNA that was purified from bacterial cultures using the DNeasy kit (QIAGEN).

### *In Vitro* Stimulation of Bone Marrow-Derived Macrophages (BMDMs)

Bone marrow cells were isolated from femur and tibia of C57/BL6 mice and were cultured in L929 fibroblast conditioned RPMI 1640 medium (containing 15% L929 supernatant and 10% FCS) for 7 days. The resulting macrophages were stimulated over night with 20 MOI of live *Hh*. Cells were pre-incubated with DATS (10 µM) 30 min before the stimulation with *Hh*. Then, the supernatants were collected for the detection of IL-6 and TNFα cytokines by ELISA.

### ELISA

IL-6 and TNFα levels in culture supernatants were evaluated using ELISA kits according to the manufacturer’s instruction (DuoSet ELISA, R&D systems, Minneapolis, MN, USA).

### Statistical Analysis

Values were expressed as mean ± SEM. Differences between experimental groups were assessed by Student’s *t*-test or one-way ANOVA, followed by Bonferroni test *P* < 0.05 was considered statistically significant: *P < 0.05; **P < 0.01; ***P < 0.001.

## Results

### MDSCs Accumulate in Spleen and in Colon of *Hh*-Infected Mice

**Figure 1:**
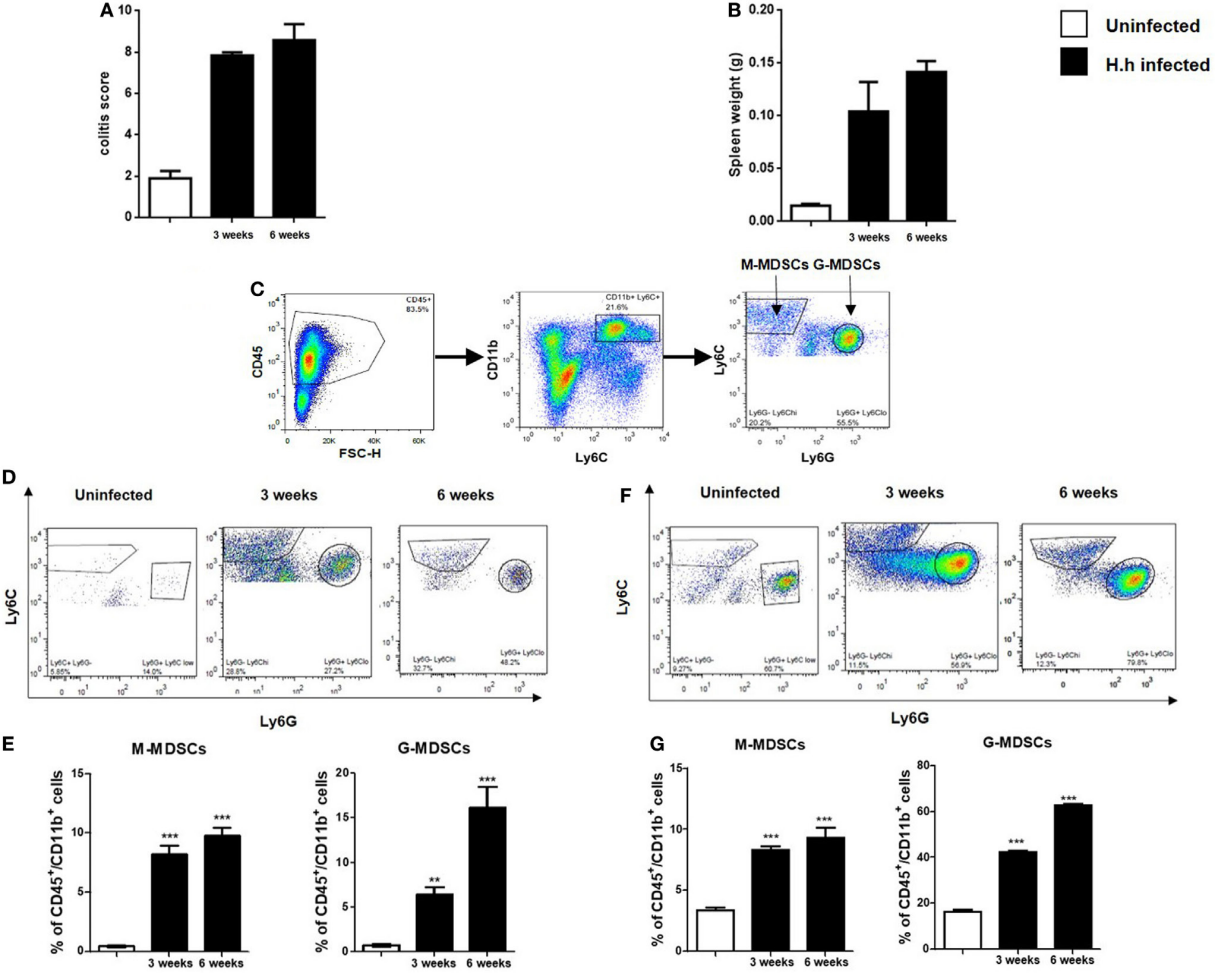
*Helicobacter hepaticus* (*Hh*) infection induces myeloid-derived suppressor cells (MDSCs) accumulation. 129SvEv *Rag2^−/−^* mice were infected with *Hh* and sacrificed after 3 or 6 weeks after infection. **(A)** Scores of colon inflammation assessed by histological analysis. **(B)** Spleen weights. **(C)** Representative FACS plots showing the gating strategy used to identify MDSCs subpopulations. Following a leukocytes gate (CD45^+^), cells were further gated for CD11b^+^ myeloid cells. Ly6C and Ly6G were used to distinguish M-MDSCs and G-MDSCs. **(D–G)** Flow cytometric analysis of M-MDSCs and G-MDSCs populations as gated in panel **(C)** and relative quantification in colon **(D,E)** and spleen **(F,G)** of 129SvEv *Rag2^−/−^* uninfected or *Hh*-infected mice. The frequency of G-MDSCs and M-MDSCs significantly increased in time-dependent manner in both colon and spleen. In particular, 6 weeks post-infection, the accumulation of G-MDSCs was about 20- and 30-fold higher in colon and spleen, respectively, as compared to uninfected mice. On the other hand, intestinal or splenic M-MDSCs fraction only increased by 10% in *Hh*-infected mice 6 weeks post-infection. Data are shown as mean ± SEM (*n* = 5 per group) (**P < 0.01, ***P < 0.001 vs. uninfected).

T cell- and B cell-deficient *Rag2^−/−^* mice were infected with *Hh*. As early as 3–6 weeks after *Hh* infection, mice developed splenomegaly and severe intestinal inflammation (Figures 1A,B). The response was dependent upon the activation of the innate immunity and characterized by a marked epithelial cell hyperplasia, extensive inflammatory infiltrates, and goblet cell depletion. *Hh* infection of *Rag2^−/−^* mice resulted in a profound proliferation of both splenic and intestinal leukocytes as previously shown (14) (data not shown). To verify the presence of MDSCs in the splenic and colon lamina propria lymphoid immune population, we performed a FACS staining for the two MDSCs subpopulation based on differential expression of Ly6C and Ly6G (Figure 1C). In fact, as previously described (9), the mononuclear fraction (M-MDSCs) is characterized by CD11b^+^Ly6G^−^Ly6C^high^ phenotype and the polimorfononuclear fraction (G-MDSCs) is characterized by CD11b^+^Ly6G^+^Ly6C^low^ phenotype. The progressive intestinal inflammation induced by *Hh* in *Rag2^−/−^* mice induced a significant (*P* < 0.001), time-dependent increase in the frequency of each fractions both in colon (Figures 1D,E) and spleen lymphoid immune populations (Figures 1F,G). Three weeks after infection, a marked expansion of both G-MDSCs and M-MDSCs was observed. Conversely, after 3 weeks more (at 6 weeks), only the G-MDSC populations resulted increased by about 20%, whereas M-MDSCs remained almost unchanged. These results suggest that the polimorfonuclear fraction might have a pivotal role in colitis development in this inflammatory setting.

### Colonic H_2_S Synthesis Is Markedly Reduced After *Hh* Infection

**Figure 2:**
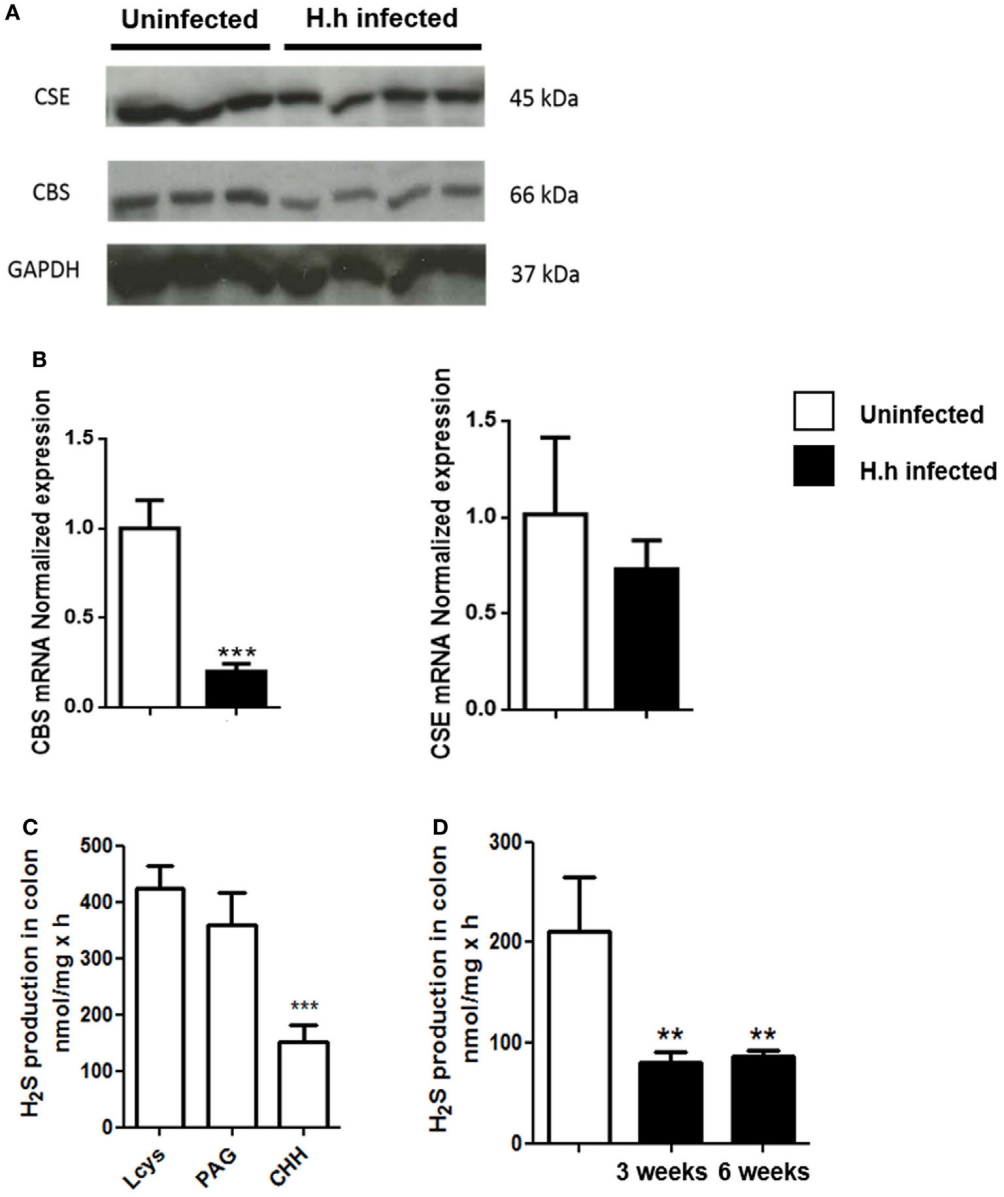
H_2_S production is significantly reduced during *Helicobacter hepaticus* (*Hh*)-induced colitis. 129SvEv *Rag2^−/−^* mice were infected with *Hh* and sacrificed 6 weeks post-infection. **(A)** Representative western blot and **(B)** relative CSE and CBS mRNA expression performed on colon samples from uninfected and *Hh*-infected mice. A significant reduction in the expression of both CBS protein and mRNA was observed in *Hh*-infected mice. **(C)** H_2_S synthesis by the colon of healthy mice was measured in colon homogenates, in the absence or presence of CBS inhibitor [(CHH), 3 mmol/L] or CSE inhibitor (PAG, 10 mmol/L). The substrate for H_2_S synthesis (l-cys) was present at 4 mmol/L. Only CHH significantly reduced H_2_S synthesis in tissue from healthy controls (***P < 0.001 vs. l-cys alone). **(D)** H_2_S levels measured in the colon of *Hh*-infected mice 3 or 6 weeks post-infection resulted significantly reduced as compared to uninfected mice. Data are shown as mean ± SEM (*n* = 5 per group) (**P < 0.01, ***P < 0.001 vs. uninfected).

Both CBS and CSE are constitutively expressed in the colon of healthy *Rag2^−/−^* mice with no significant difference between CBS/CSE protein (Figure 2A) and gene expression (Figure 2B). Thus, we evaluated the metabolic activity of these enzymes by measuring their ability to produce sulfide adding the substrate l-cys and their respective inhibitors. In healthy mice, we found that the preferential CBS inhibitor CHH (3 mmol/L) reduced H_2_S synthesis by ~65% (*P* < 0.001, vs. l-cys), whereas the selective CSE inhibitor PAG (10 mmol/L) was ineffective (Figure 2C). These results imply that in the colon of *Rag2^−/−^* mice: (i) CBS appears to be the major responsible for colonic H_2_S synthesis and (ii) change in the CBS activity accounts for H_2_S production. After 3 and 6 weeks following *Hh* mouse infection, we observed a significant reduction in the expression of both CBS protein and mRNA (*P* < 0.01 and *P* < 0.001 vs. uninfected, respectively; Figures 2A,B). This effect translated into a significant reduction of H_2_S levels in inflamed colon (Figure 2D). CSE levels were left unchanged (Figures 2A,B).

### H_2_S Donor Reduces Colitis Severity

**Figure 3:**
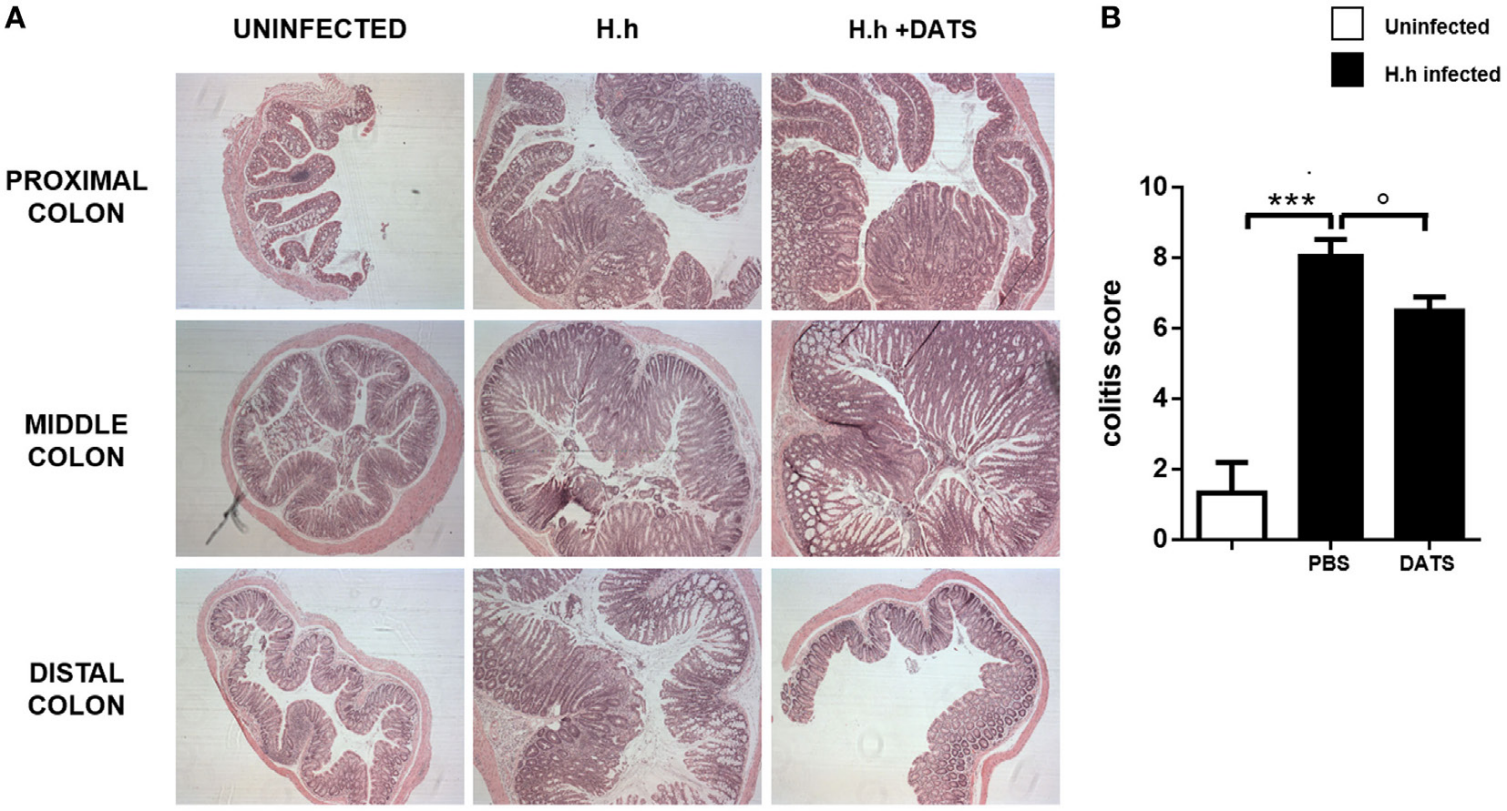
H_2_S donor reduces the severity of colitis. **(A)** Representative photomicrographs (magnification ×50) of H&E-stained proximal, middle, and distal colon isolated from healthy mice and from 6 weeks *Helicobacter hepaticus*-infected mice treated with DATS or only vehicle (PBS) and correspondent **(B)** inflammation score. DATS significantly reduced inflammation in the colon. Data are shown as mean ± SEM (*n* = 5 per group) (***P < 0.001 vs. uninfected. °*P* < 0.05 vs. PBS).

**Figure 4:**
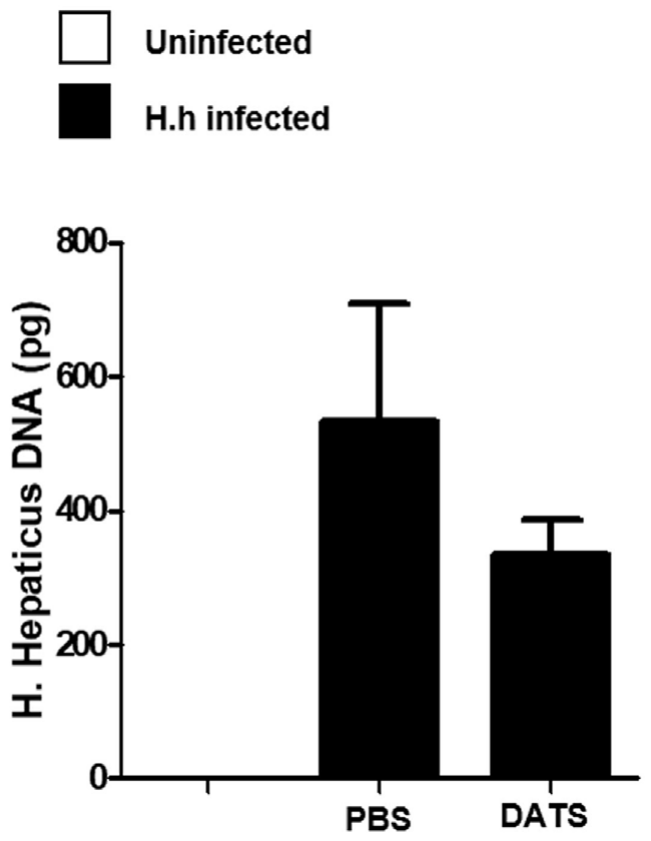
DATS does not affect *Helicobacter hepaticus* (*Hh*) colonization levels. Quantification of *Hh* DNA in cecum content samples from healthy mice and from 6 weeks *Hh*-infected mice treated with DATS or vehicle (PBS) by using a real-time PCR assay. Data are shown as mean ± SEM (*n* = 5 per group).

**Figure 5:**
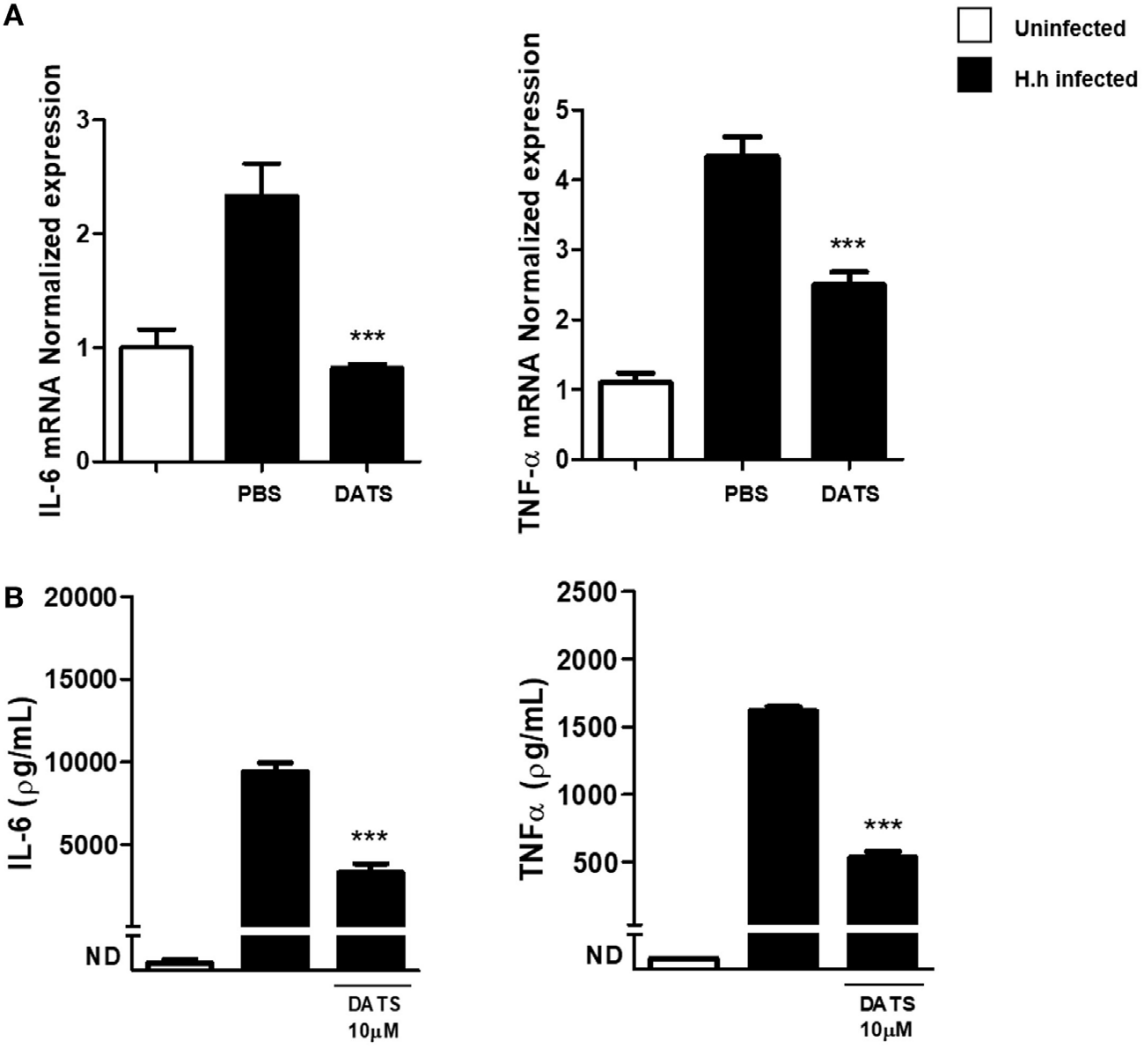
DATS reduces pro-inflammatory cytokines production. **(A)** Relative expression of TNF-α and IL-6 mRNA within the colons of both uninfected mice and *Helicobacter hepaticus* (*Hh*)-infected mice, 6 weeks post-infection, receiving DATS or vehicle (PBS). Expression of cytokines mRNA was determined by real-time quantitative PCR. Data are shown as mean ± SEM (*n* = 5 per group) (***P < 0.001 vs. PBS). **(B)** Bone marrow-derived macrophages from uninfected mice were stimulated with live *Hh* (20 MOI). Incubation of cells with DATS (10 µM) significantly reduced IL-6 and TNFα release. Data are shown as mean ± SEM of three pooled independent experiments (***P < 0.001 vs. *Hh*-infected).

To support the hypothesis that the reduction of H_2_S synthesis contributes to the development of *Hh*-induced colitis, we performed a pharmacological modulation. DATS, a natural long lasting H_2_S donor (22) was orally administered to *Rag2^−/−^* mice previously infected with *Hh* (1.0 × 10^8^ CFU). Four weeks after infection, a group of mice received DATS at 50 mg/kg while the control group received only the vehicle (PBS) every day for 2 weeks. Mice were analyzed for intestinal inflammation 6 weeks after the first infection with *Hh*. In DATS-treated group, a significant (*P* < 0.05) reduction of colon inflammation as compared with the control group was evident. The histological analysis revealed that the reduction in colon inflammation score induced by DATS was more marked in the colon distal part (Figures 3A,B). The anti-inflammatory effect of DATS was not associated with a change in bacterial burden, as we could not detect any differences in cecal *Hh* colonization levels upon treatment (Figure 4). Thus, to gain insights into the anti-inflammatory mechanism(s) of H_2_S in this model of colitis-associated cancer, we analyzed the content of pro-inflammatory cytokines. As shown in Figure 5A, the inflammatory response was sustained by high levels of IL-6 and TNF-α mRNA expression in colonic tissues from infected mice, as compared to uninfected mice. In DATS-treated mice, a reduction of the pro-inflammatory cytokines mRNA was observed confirming a key role for these cytokines. This hypothesis is further supported by the *in vitro* experiments carried out on BM-derived macrophages (BMDMs) stimulated with *Hh* bacteria (20 MOI). As expected, infection of BMDMs cells with *Hh* induced a sustained release of IL-6 and TNFα in the supernatants as compared to unstimulated BMDM. Overnight incubation of infected BMDMs cells with DATS (10 µM) significantly (*P* < 0.001) reduced the release of these cytokines (Figure 5B).

### H_2_S Reduces the Number of G-MDSCs

**Figure 6:**
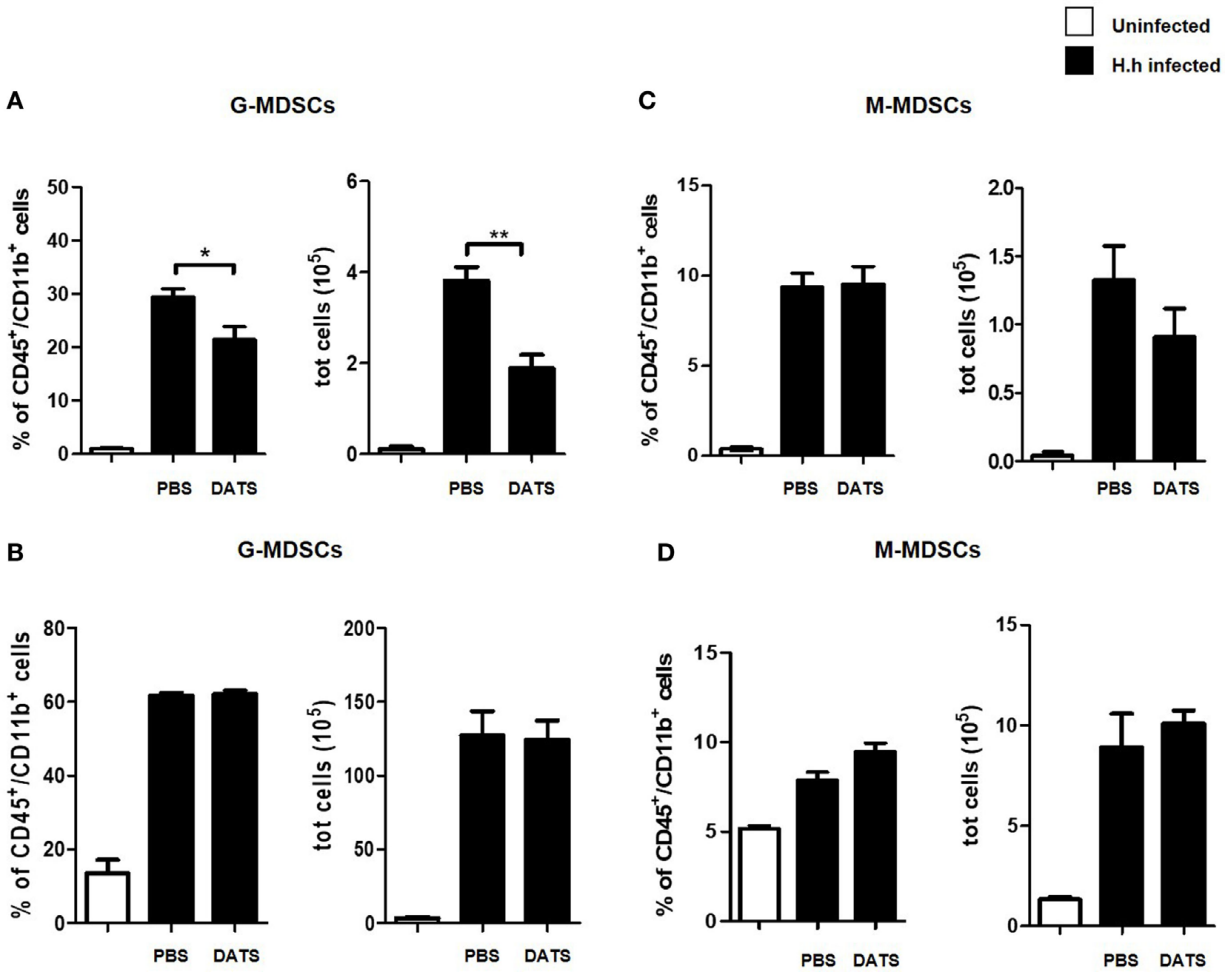
DATS inhibits G-MDSCs accumulation in the colon of *Helicobacter hepaticus* (*Hh*)-infected mice. Leukocytes were isolated from the colon and the spleen of 129SvEv *Rag2^−/−^* uninfected or *Hh*-infected 6 weeks post-infection. Infected mice were treated with DATS or vehicle (PBS). G-MDSCs and M-MDSCs frequencies and absolute number were assessed by flow cytometry in colon **(A,C)** and in spleen **(B,D)**. G-MDSCs frequency and number was reduced in the colon following treatment with DATS. Data are shown as mean ± SEM (*n* = 5 per group) (*P < 0.05, **P < 0.01, DATS vs. PBS).

To evaluate if the hydrogen sulfide effect on the intestinal inflammation was due to a modification of the innate immune response, we isolated and characterized colon and spleen leukocytes from *Hh*-infected mice. As expected, *Hh*-mediated inflammation was associated with an increase in the frequency of both G-MDSCs and M-MDSCs cells in infected mice as compared with uninfected mice. Replenishing hydrogen sulfide, by using as exogenous source the H_2_S donor DATS, induced a significative reduction both in the frequency (*P* < 0.05 vs. PBS) and in the absolute number (*P* < 0.01 vs. PBS) of G-MDSCs in colon (Figure 6A). It has to be noted that hydrogen sulfide addition did not affect G-MDSCs frequencies in the spleen (Figure 6B) suggesting a localized anti-inflammatory effect. Finally, the number of both colon and spleen M-MDSCs were not affected by DATS treatment (Figures 6C,D). Thus, the H_2_S-induced reduction of *Hh*-triggered inflammation is related to the modulation of the innate component of the immune system.

## Discussion

Infiltration of immune cells, specifically CD11b^+^ myeloid cells, and their aberrant activation play a central role in carcinogenesis contributing to create a pro-inflammatory microenvironment that enables tumor promotion (23). MDSCs have emerged as key effector cells in tumor microenvironment responsible for tumor progression and metastasis (24). Since pro-inflammatory molecules induce MDSCs, we speculate that *Hh* infection of *Helicobacter*-free 129SvEv/*Rag2^−/−^* mice could promote MDSC accumulation in colon and spleen facilitating tumor formation. Conflicting data are present in literature regarding MDSCs accumulation and function during colitis in mice. These divergences are likely due to the specific experimental animal model used. Haile et al., using a model of T cell-mediated autoimmune enterocolitis, showed that MDSCs were protective and suppressed development of disease (25). In another study, Zhang and coworkers, using a model of colitis induced by dextran sodium sulfate (DSS), demonstrated that the transfer of splenic DSS-derived CD11b^+^Gr-1^+^ MDSCs into a recipient mice suppressed myeloid-lineage cell development in the lamina propria and ameliorated disease parameters (26). There is also evidence supporting a pro-inflammatory role of myeloid cells in experimental IBD. In fact, Guan et al. observed that both CD11b^+^Ly6C^+^ and CD11b^+^Ly6G^+^ cells increased in spleen and in colonic lamina propria in mice with acute colitis and also correlated with the intestinal inflammation severity (27).

Thus, to better define the role of MDSCs in colitis-associated cancer development, we decided to use an innate immune-mediated IBD model lacking the adaptive immune response. Another benefit of the model chosen is that it closely resembles human IBD since members of the Helicobacteraceae family have been found in the colon of IBD patients (28, 29). The development of colitis in *Hh*-infected *Rag2^−/−^* mice was accompanied by an increase of MDSCs in lymphoid and non-lymphoid tissue in a time-dependent manner. The G-MDSCs subtype was predominant both in the spleen and in the colon of infected mice. This finding is in accordance with the current relevant literature and confirms that during tumor progression the G-MDSC population is predominant (30). Oxidative stress in human IBD correlates with disease activity and represents one of the key features of tumor initiation (31). More recently, a role for hydrogen sulfide in oxidative stress has been defined (32). H_2_S is an endogenous mediator that exhibits several anti-inflammatory activities and contribute to gastric mucosal defense (15). Its protective role in the resolution of colitis and ulcer healing in rats and mice has been elegantly demonstrated by Wallace and coworkers (15, 16, 33, 34). In the attempt to better define the downstream signaling in this model we have assessed the possible role played by the hydrogen sulfide pathway. It is widely assumed that desulfhydration of l-cys is the major source of H_2_S in mammals and is catalyzed by the *trans*-sulfuration pathway enzymes: CBS and CSE. They are both P5P-dependent lyases and generate H_2_S in many tissues including brain, liver, kidney, ileum, uterus, and placenta. Beside other main reactions, CBS and CSE catalyze the synthesis of cystathionine from l-cysteine and l-homocysteine, generating H_2_S (35). In addition, a third pathway represented by the enzymes 3-mercaptopyruvate sulphurtransferase (3MST) in conjunction with cysteine aminotransferase, has been found to contribute to H_2_S production in the brain and in the vascular endothelium of thoracic aorta (36, 37). Finally, very recently, it has been discovered that also cysteinyl-tRNA synthetases 2 (CARS2), a mitochondrial isoform of CARS, are involved in polysulfide production (38). CBS and CSE are currently considered the major H_2_S enzymatic source in a variety of tissues; we therefore concentrated our attention on these enzymes. However, the possible contribution of the other alternative metabolic pathways in the development of colitis in *Hh*-infected mice cannot be completely excluded. Our results demonstrated that CBS is the primary H_2_S-producing enzyme in the colon of both healthy and infected Rag2^−/−^ mice. In this regard, CBS in rat colon do represent the major source of colonic H_2_S either in healthy state or during inflammation (16). H_2_S synthesis by the *Hh*-infected mice colon was reduced by 100-fold after induction of colitis at 3 and 6 weeks, thereby implying a putative protective role for hydrogen sulfide. This reduction of H_2_S synthesis well fits with the downregulation of CBS protein and mRNA expression in the colon of infected mice during colitis. *Hh*-mediated inflammation induced an increase in the frequency of G-MDSCs and M-MDSCs cells in colon and in spleen of infected mice as opposite to control mice. To further investigate on the contribution of hydrogen sulfide, we designed an experiment where the exogenous hydrogen sulfide was “furnished” by mean of a donor. Chronic oral administration of the H_2_S donor DATS did not cause eradication of bacteria or decreased levels of colonization in mice but reduced colon inflammation. It has to be noted that the mechanisms by which H_2_S is released from DATS have in depth analyzed by several research groups (22, 39–42). In particular, it has been shown that H_2_S is liberated from DATS in presence of GSH and l-cys (43). However, sulfane sulfur (polysulfides), rather than H_2_S, are considered the active agent in physiological signaling (44–47). The recent study (38) showing that CBS and CARS2 can also produce sulfane sulfur species directly from cystine and cysteine, respectively, added another tassel in this already very complicated puzzle.

Finally, in order to gain mechanistic insights into this anti-inflammatory effect, we have evaluated changes in the inflammatory phenotype of the immune population in the colon. Treatment with DATS induced a significative reduction both in the frequency and in the absolute number of G-MDSCs in colon without affecting G-MDSCs frequencies in the spleen. This finding implies a localized selective effect of hydrogen sulfide on colon G-MDSCs population.

It has been shown that *Hh*-induced malignancy in *Rag2^−/−^* mice was readily reversible by blocking underlying bacteria-driven inflammation with antibodies directed at TNF-α and IL-6 (48, 49). TNF-α and IL-6 signaling has been proposed as a tumor-promoting mechanism in colitis-associated cancer. In fact, the levels of these pro-inflammatory cytokines increase during inflammatory reactions and are responsible, at least in part, for MDSCs accumulation and for the increase in their suppressive activity (24). In tune with these findings, levels of IL-6 and TNF-α mRNA are increased in colonic tissues from infected mice. Interestingly, hydrogen sulfide replenishment with DATS reduced TNF-α and IL-6 levels as compared to control mice. To further support our hypothesis, we performed experiments on BM-derived macrophages stimulated with *Hh* bacteria. Indeed, it is known that *Hh* challenge of *ex vivo*-cultured BMDMs induces activation of both the NF-κB and ERK pathways (50, 51). Infection of BMDMs cells with *Hh* induced a sustained release of the pro-inflammatory cytokines IL-6 and TNFα in the supernatants as compared to unstimulated BMDM. Treatment of BMDMs cells with exogenous hydrogen sulfide, by using DATS, reduced cytokines release mimicking the *in vivo* setting. In conclusion, these results indicate that the metabolic pathway l-cys/H_2_S can exert a protective role in intestinal *Hh-*induced inflammation. Dysregulation of H_2_S homeostasis has also been implicated in numerous pathological conditions and diseases (52). Thus, the reduction of H_2_S levels reported during colitis might induce metabolic changes in the microenvironment, as the alteration of the redox cellular status, which promotes tumorigenesis. Metabolic reprograming has been suggested as a key hallmark of cancer progression (53). Recent studies have revealed that immune cells possess distinct metabolic characteristics that influence their immunological phenotype and functions and so their contribution to cancer progression (54). Thus, the identification of new metabolic targets could be of great importance in modifying the plasticity of tumor-promoting immune cells and in CRC prevention. Our results demonstrating that MDSCs mediate significant intestinal inflammation upon stimulation with pathogenic *Hh* assume a therapeutic significance since, targeting MDSCs would be promising treatment option for IBD patient to reduce the risk of CRC and to manage inflammatory symptoms in order to provide an improved quality of life.

## Ethics Statement

Experiments were conducted in accordance with the UK Scientific Procedures Act (1986) under a Project License (PPL) authorized by the UK Home Office Animal Procedures Committee and approved by the Sir William Dunn School Ethical Review Committee.

## Author Contributions

PC designed, performed the experiments, and analyzed the data; TS performed the experiments; GC revised critically the manuscript; KM and AI supervised all the experiments, revised critically the intellectual contributions to the manuscript, and gave final approval to the publication.

## Conflict of Interest Statement

The authors declare that the research was conducted in the absence of any commercial or financial relationships that could be construed as a potential conflict of interest.

## Footnotes

^1^Available from: http://globocan.iarc.fr/Pages/fact_sheets_cancer.aspx?cancer=colorectal (Accessed: October 25, 2017)
